# MLK3 is a novel target of dehydroglyasperin D for the reduction in UVB-induced COX-2 expression *in vitro* and *in vivo*

**DOI:** 10.1111/jcmm.12311

**Published:** 2014-08-30

**Authors:** Sung Keun Jung, Su Jeong Ha, Yeong A Kim, Jihoon Lee, Tae-Gyu Lim, Yun Tai Kim, Nam Hyouck Lee, Jun Seong Park, Myeong-Hun Yeom, Hyong Joo Lee, Ki Won Lee

**Affiliations:** a WCU Biomodulation Major, Department of Agricultural Biotechnology and Center for Food and Bioconvergence, Seoul National UniversitySeoul, Korea; b Division of Metabolism and Functionality Research, Korea Food Research InstituteSeongnam, Korea; c Skin Research Institute, Amorepacific R&D CenterYongin, Korea; d Research Institute of Bio Food Industry, Institute of Green Bio Science and Technology, Seoul National UniversityPyeongchang, Korea; e Advanced Institutes of Convergence Technology, Seoul National UniversitySuwon, Korea

**Keywords:** dehydroglyasperin D, cyclooxygenase-2, mixed-lineage kinase 3, licorice, inflammation

## Abstract

Dehydroglyasperin D (DHGA-D), a compound present in licorice, has been found to exhibit anti-obesity, antioxidant and anti-aldose reductase effects. However, the direct molecular mechanism and molecular targets of DHGA-D during skin inflammation remain unknown. In the present study, we investigated the effect of DHGA-D on inflammation and its mechanism of action on UVB-induced skin inflammation in HaCaT human keratinocytes and SKH-1 hairless mice. DHGA-D treatment strongly suppressed UVB-induced COX-2 expression, PGE2 generation and AP-1 transactivity in HaCaT cells without affecting cell viability. DHGA-D also inhibited phosphorylation of the mitogen-activated protein kinase kinase (MKK) 3/6/p38, MAPK/Elk-1, MKK4/c-Jun N-terminal kinase (JNK) 1/2/c-Jun/mitogen, and stress-activated protein kinase (MSK), whereas phosphorylation of the mixed-lineage kinase (MLK) 3 remained unaffected. Kinase and co-precipitation assays with DHGA-D Sepharose 4B beads showed that DHGA-D significantly suppressed MLK3 activity through direct binding to MLK3. Knockdown of MLK3 suppressed COX-2 expression as well as phosphorylation of MKK4/p38 and MKK3/6/JNK1/2 in HaCaT cells. Furthermore, Western blot assay and immunohistochemistry results showed that DHGA-D pre-treatment significantly inhibits UVB-induced COX-2 expression *in vivo*. Taken together, these results indicate that DHGA-D may be a promising anti-inflammatory agent that mediates suppression of both COX-2 expression and the MLK3 signalling pathway through direct binding and inhibition of MLK3.

## Introduction

Cyclooxygenase (COX)-2 is an inducible enzyme that regulates prostanoid synthesis, lipid peroxidation and prostaglandins (PGs), and has been implicated in the acceleration of inflammation and carcinogenesis [1,2]. Multiple lines of evidence have shown that acute and chronic exposure to ultraviolet (UV) irradiation can induce abnormal expression of COX-2, and subsequently lead to the development of skin cancer [3–5]. *Cox-2* knockout mice are protected against UV-induced skin carcinogenesis [6], while overexpression of COX-2 has been reported to enhance skin cancer development in transgenic mice [6,7]. Pharmacological studies have revealed that treatment with non-steroidal anti-inflammatory drugs and COX-2 inhibitors such as celecoxib prevents the onset of UV-induced skin cancer [6,7]. Our previous studies have suggested that suppression of UV-induced COX-2 expression using natural phytochemicals may represent a promising strategy for the prevention of skin carcinogenesis [3–5].

Mixed-lineage kinases (MLK) constitute a family of serine/threonine protein kinases that are implicated in multiple signalling cascades involving mitogen-activated-protein kinases (MAPKs) [8]. UV irradiation escalates MLK3 activity and subsequently enhances activator protein (AP)-1 transcriptional activity by increasing the phosphorylation of c-Jun amino-terminal kinase (JNK)/c-Jun [8]. The targeted disruption of *Mlk3* (*Mlk3*^*−/−*^) can result in selective reduction in JNK activation without embryonic lethality in response to tumour necrosis factor (TNF) treatment [9]. CEP-11004, a specific MLK3 inhibitor, significantly suppresses ceramide-induced JNK activation without affecting the activity of p38 or extracellular signal-regulated kinase (ERK) [10]. However, the mechanism of MLK3 in UVB-induced inflammation and a potential clinically suitable inhibitor of the kinase have yet to be identified.

The consumption of natural phytochemicals has been linked to the prevention or delay of cancer development [11,12]. Compounds in licorice roots have been shown to exhibit various biological activities, including antioxidant, anti-inflammatory and anti-cancer effects [13–15]. Several active compounds in licorice, including glycyrrhetinic acid, licochalcone A, isoangustone A and isoliquiritienin have been demonstrated to possess anti-cancer effects [13,16–18]. Dehydroglyasperin D (DHGA-D) is a recently identified anti-obesity component in licorice, acting as a ligand for the peroxisome proliferator-activated receptor-γ [19]. Although DHGA-D was recently shown to exhibit antioxidant and aldose reductase inhibitory activities [16,20], the mechanism of action of its inhibitory effect on COX-2 expression remains unknown.

In the present study, we investigated the effects of DHGA-D on UVB-induced COX-2 signalling pathways in HaCaT human keratinocytes. We observed that DHGA-D inhibited UVB-induced COX-2/PGE2 production. Furthermore, DHGA-D exhibited inhibitory effects on UVB-induced AP-1 transactivation as well as MKK3/6 and MKK4/7 signalling pathways because of suppression of MLK3 activity through direct binding. These findings suggest that DHGA-D may be a potent anti-inflammatory agent that inhibits COX-2 expression through the direct suppression of MLK3 activity.

## Materials and methods

### Materials

DMEM, gentamicin and L-glutamine were obtained from Gibco–BRL (Carlsbad, CA, USA). Foetal bovine serum (FBS) was purchased from Gemini Bio-Products (Calabasas, CA, USA). Antibodies to detect phosphorylated p38 (Tyr180/Tyr182), total p38, phosphorylated ElK-1 (ser383), total Elk-1, phosphorylated JNK (Thr183/Tyr185), total JNK, phosphorylated c-Jun (ser63), phosphorylated MKK3/6 (Ser189/207), total MKK3, phosphorylated MKK4 (Ser257/Thr261) and phosphorylated MSK1 (Ser376) were purchased from Cell Signaling Biotechnology (Beverly, MA, USA). Phosphorylated MLK3 protein (Thr277/Ser281) was obtained from Upstate Biotechnology (Lake Placid, NY, USA). ATP and the chemiluminescence detection kit were purchased from Amersham Pharmacia Biotech (Piscataway, NJ, USA), and the protein assay kit was obtained from Bio-Rad Laboratories (Hercules, CA, USA). G418, luciferase assay substrate and MTS solution were purchased from Promega (Madison, WI, USA). The PGE2 enzyme immunoassay kit was obtained from Cayman Chemical (Ann Arbor, MI, USA). DHGA-D was kindly provided by Dr. SS Lim [21].

### Cell culture and viability assay

Human epidermal keratinocyte HaCaT cells were maintained in DMEM containing 10% FBS (Atlanta Biologicals, Lawrenceville, GA, USA), 100 U/ml of penicillin and 100 mg/ml of streptomycin at 37°C in a 5% CO_2_ humidified incubator. The UVB light source (Bio-Link Crosslinker; Vilber Lourmat, Marne-la-Vallée, France) emitted wavelengths of 254, 312, and 365 nm, with peak emission at 312 nm. To estimate cell viability, HaCaT cells were seeded (10^3^ cells/well) in 96-well plates and incubated at 37°C in a 5% CO_2_ incubator. After the cells were treated with DHGA-D, 100 μl of MTS solution in the presence of phenazine methosulphate was added to each well. After 1 hr of incubation, the absorbance levels for formazan at 490 and 690 nm were measured by using a microplate reader.

### Animals

The animal experimental protocol (SNU-120614-3) was approved and animals were maintained under specific pathogen-free conditions based on the guidelines established by the Institutional Animal Care and Use Committee of Seoul National University. Female SKH-1 hairless mice (5 week old; mean bw, 25 g) were purchased from the Institute of Laboratory Animal Resources at Seoul National University. Animals were acclimated for 1 week prior to the study and had free access to food and water. The animals were housed in climate-controlled quarters (24°C at 50% humidity) with a 12-hr light/dark cycle.

### PGE2 assay

DHGA-D was treated to HaCaT cells plated in 6-well dishes at 80% confluency, 1 hr prior to UVB (0.05 J/cm^2^) irradiation, and then harvested 18 hrs later. The quantity of PGE2 released into the medium was measured by using a PGE2 enzyme immunoassay kit (Cayman Chemical Company, Ann Arbor, MI, USA).

### Luciferase assay for AP-1 transactivation

HaCaT cells (8 × 10^3^ total) transfected with AP-1 luciferase plasmid [22] were added to each well of 96-well plates. Plates were incubated at 37°C in a 5% CO_2_ incubator. When cells reached 80–90% confluence, they were starved by culturing in serum-free DMEM for a further 24 hrs. Cells were then treated with DHGA-D (2.5 and 5 μM) for 1 hr prior to UVB (0.05 J/cm^2^) exposure and then incubated for 5 hrs. Cells were then disrupted with 100 μl of lysis buffer [0.1 M potassium phosphate buffer (pH 7.8), 1% Triton X-100, 1 mM dithiothreitol (DTT) and 2 mM EDTA], after which luciferase activity was measured by using a luminometer (Luminoskan Ascent; Thermo Electron, Helsinki, Finland).

### Western blot assay

For Western blot assay, cells (1.5 × 10^6^ total) were cultured in a 10 cm dish for 48 hrs, followed by starvation in serum-free DMEM for 24 hrs. Cells were then treated with DHGA-D (2.5 or 5 μM) for 1 hr and irradiated with UVB (0.05 J/cm^2^). The protein concentration was determined by using a dye-binding protein assay kit (Bio-Rad Laboratories) following instructions in the manufacturer's manual. Lysate protein was subjected to 10% SDS-PAGE and transferred to a polyvinylidene difluoride membrane (Amersham Pharmacia Biotech). After transferring, the membranes were incubated with specific primary antibodies at 4°C overnight. Protein bands were visualized by using a chemiluminescence detection kit (Amersham Pharmacia Biotech) after hybridization with a horseradish peroxidase-conjugated secondary antibody.

### MLK3 kinase assay

MLK3 kinase activity was assayed in accordance with the instructions provided by Upstate Biotechnology (Billerica, MA, USA). Exactly 20 ng of MLK3 protein was added to a mixture containing myelin basic protein, 5× assay buffer, and diluted [γ-32P]ATP solution with or without DHGA-D. Reactions were carried out at 30°C for 10 min., and incorporated radioactivity was determined by using a scintillation counter. The kinase activity data represent the mean of three independent experiments.

### Co-precipitation assays

CNBr-activated DHGA-D beads were produced as described in a previous study [5]. For the co-precipitation assay, 100 ng of recombinant active MLK3 was incubated with DHGA-D-conjugated or non-DHGA-D-conjugated Sepharose 4B beads (100 μl, 50% slurry) in reaction buffer [50 mM Tris (pH 7.5), 5 mM EDTA, 150 mM NaCl, 1 mM dithiothreitol, 0.01% Nonidet P-40, 2 μg/ml of bovine serum albumin (BSA), 0.02 mM PMSF, and 1 μg of protease inhibitor mixture]. After washing the beads, bound proteins were analysed by Western blot assay by using the antibodies described above.

### Knockdown of MLK3

For knockdown of MLK3, HaCaT cells were transfected with scrambled sequence or 3 μg human MLK3 siRNA (derived from the sequence 5′-GGGCAGTGACGTCTGGAGTTT-3′, nucleotides 903-923 of the cDNA sequence) plasmids by using Lipofectamine 2000 (Invitrogen, Carlsbad, CA, USA), following the manufacturer's suggested protocols. The transfected cells were then exposed to UVB irradiation and used in subsequent experiments.

### Immunohistochemical analysis

Sections (5-μm thick) of 10% neutral formalin solution-fixed, paraffin-embedded tissues were cut on silane-coated glass slides. Deparaffinized sections were then incubated in proteinase K (20 μg/ml) for 20 min. at room temperature. In addition, the sections were heated for 15 min. in 10 mM citrate buffer (pH 6.0) in a microwave oven for antigen retrieval. For the detection of target proteins, slides were incubated with affinity-purified primary antibody in a refrigerator overnight in 1% BSA solution and then developed by using the HPR En VisionTM System (DAKO, Carpinteria, CA, USA) for anti-rabbit and antimouse antibodies. Peroxidase-binding sites were detected by staining with 3,3′-diaminobenzidine tetrahydrochloride (Dako). Finally, counterstaining was performed by using Mayer's haematoxylin.

### Statistical analysis

Where appropriate, data are expressed as the mean ± SEM, and significant differences were determined by using one-way anova. A probability value of *P* < 0.05 was used as the criterion for statistical significance.

## Results

### DHGA-D inhibits UVB-induced COX-2 expression, PGE2 generation and AP-1 transactivity in HaCaT cells

**Fig. 1:**
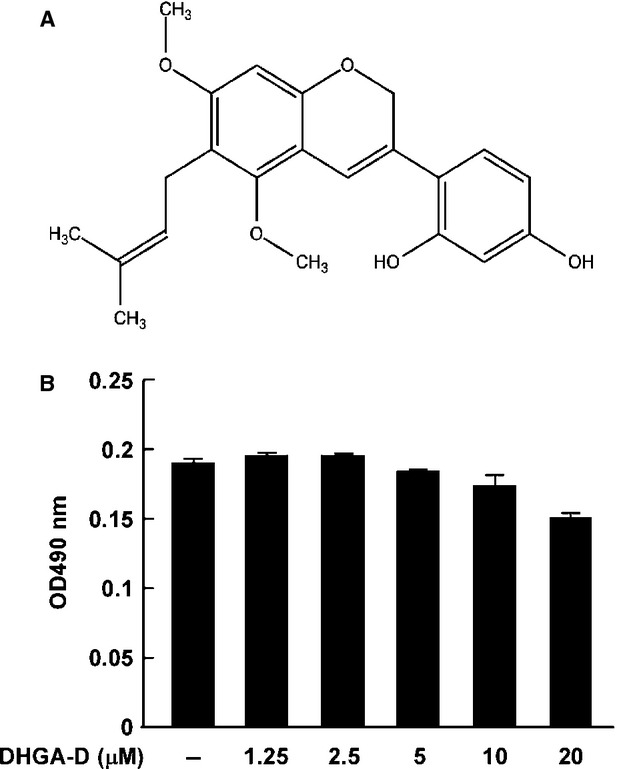
Effect of DHGA-D on HaCaT cell viability. (**A**) Structure of DHGA-D. (**B**) DHGA-D exhibits no detectable cell cytotoxicity up to 20 μΜ in human HaCaT cells. Cell viability was measured by MTS assay as described in Materials and Methods. Data are presented as the mean ± SD of three independent experiments.

**Fig. 2:**
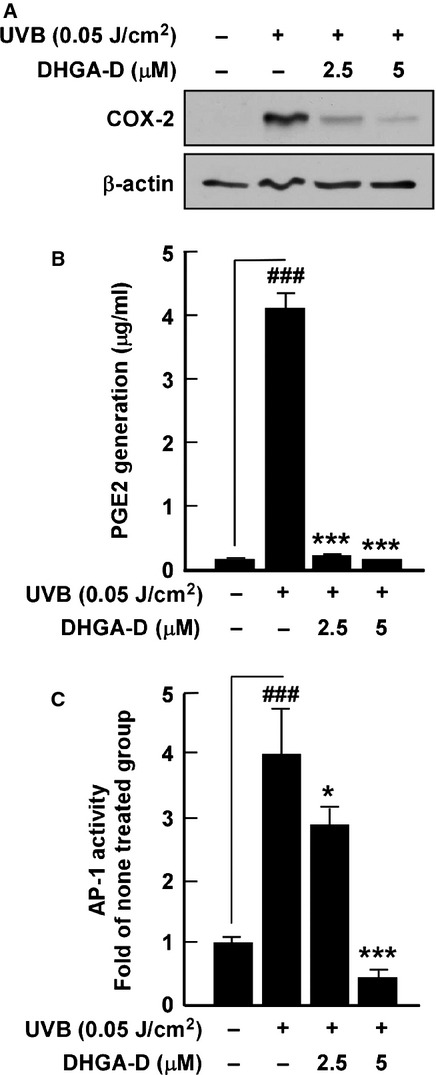
Effect of DHGA-D on UVB-induced COX-2 expression, PGE2 generation and AP-1 transactivity in HaCaT cells. (**A**) DHGA-D inhibits UVB-induced COX-2 expression in HaCaT cells. Expression levels of COX-2 and β-actin were determined by Western blot assay. Data are representative of three independent experiments that gave similar results. (**B**) DHGA-D inhibits UVB-induced PGE2 generation in HaCaT cells. Expression levels of PGE2 were determined by using a Prostaglandin E2 Express EIA kit (Cayman Chemical Company) following the manufacturer's instructions. The quantity of PGE2 is presented as the mean ± SD of three independent experiments. (**C**) DHGA-D suppresses UVB-induced AP-1 transactivity in HaCaT cells. For the luciferase assay, HaCaT cells were stably transfected with an AP-1-luciferase reporter plasmid and cultured as described in the Materials and Methods. AP-1 luciferase activity is presented as the mean ± SD of three independent experiments. Hash symbols (###) indicate a significant difference (*P* < 0.001) between the control group and the group exposed to UVB alone; asterisks (*) and (***) indicate significant differences [(*P* < 0.05) and (*P* < 0.001), respectively] between groups irradiated with UVB and DHGA-D and the group exposed to UVB alone.

Ultraviolet irradiation has been reported as inducing abnormal COX-2 expression [3–5] and un-regulated COX-2 mediates skin inflammation and carcinogenesis [6,7,23]. Thus, we sought to investigate whether DHGA-D (Fig. 1A) could inhibit UVB-induced COX-2 expression. No evidence of cytotoxicity was observed at the indicated concentrations (Fig. 1B). Western blot assay showed that DHGA-D strongly suppressed UVB-induced COX-2 expression in HaCaT cells (Fig. 2A). PGE2, a product of COX-2, is closely related to inflammation by mediating inflammatory signalling pathways. PGE2 quantification clearly showed that DHGA-D completely suppressed UVB-induced PGE2 generation in HaCaT cells (Fig. 2B). AP-1, a major transcription factor that regulates COX-2 expression, plays a vital role in carcinogenesis and is a promising chemopreventive target [24,25]. In HaCaT cells stably transfected with an AP-1-luciferase reporter plasmid, DHGA-D significantly inhibited UVB-induced AP-1 transactivation in a dose-dependent manner (Fig. 2C).

### DHGA-D inhibits MKK3/6 and MKK4 signalling pathways in HaCaT cells

**Fig. 3:**
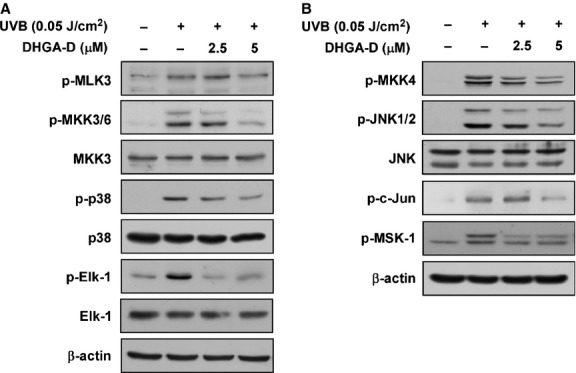
Effect of DHGA-D on UVB-induced phosphorylation of MAPKs in HaCaT cells. (**A**) and (**B**) DHGA-D inhibits UVB-induced phosphorylation (*p*-) of MKK3/6, p38, Elk-1, MKK4, JNK1/2, c-Jun, and MSK1 in HaCaT cells. Cells were pre-treated with DHGA-D at the indicated concentrations (2.5 and 5 μM) for 1 hr, stimulated with UVB irradiation, and harvested 15 or 30 min. later. Phosphorylation was detected by Western blotting assay with specific antibodies. Data are representative of three independent experiments that gave similar results.

Published studies have reported that *cox-2* is a target gene with an AP-1-binding site in its promoter region, while AP-1 activity is regulated by activation of MAPK signalling pathways [26,27]. To further assess which signalling pathway was affected by DHGA-D, we examined the effect of the compound on UVB-induced phosphorylation of MAPKs in HaCaT cells. Based on Western blot analysis, DHGA-D inhibited UVB-induced phosphorylation of MKK3/6, p38, Elk-1, MKK4, JNK1/2, c-Jun, and MSK-1 in HaCaT cells (Fig. 3A and B).

### DHGA-D inhibits MLK3 activity through direct binding

**Fig. 4:**
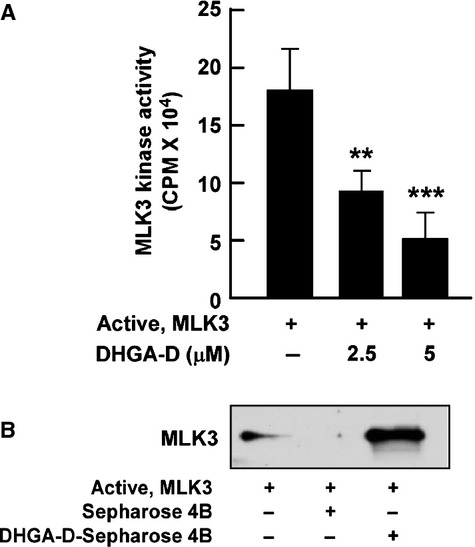
Effect of DHGA-D on MLK3 activity and binding. (**A**) DHGA-D suppresses MLK3 activity. MLK3 kinase assays were performed as described in the Materials and Methods. Data are presented as the means ± SD of three independent experiments. Asterisks [(**) and (***)] indicate significant differences [(*P* < 0.01) and (*P* < 0.001), respectively] between groups with or without DHGA-D. (**B**) DHGA-D directly binds to MLK3. Binding affinity between DHGA-D and MLK3 was confirmed *in vitro* by immunoprecipitation assay with DHGA-D Sepharose beads, followed by immunoblotting by using an anti-MLK3 antibody as described in the Materials and Methods. First lane: input control, MLK3 protein; second lane: (control), MLK3 protein precipitated with Sepharose 4B beads; third lane: MLK3 protein precipitated with DHGA-D Sepharose 4B beads. Each experiment was performed three times.

Previous studies showed that MLK directly regulates MKK3/6 and MKK4 phosphorylation [28,29]. Additionally, despite the significant inhibition of UVB-induced MKK3/6 and MKK4 signalling pathways, DHGA-D did not inhibit MLK phosphorylation. Therefore, we hypothesized that DHGA-D may directly inhibit MLK3 activity. We investigated this possibility by using active MLK3 protein. MLK3 kinase assay revealed that DHGA-D significantly inhibited MLK3 activity (Fig. 4A). Furthermore, immunoprecipitation using DHGA-D-conjugated Sepharose beads showed that DHGA-D bound directly to MLK3 (Fig. 4B).

### Knockdown of MLK3 inhibits UVB-induced COX-2 expression and the MLK3 signalling pathway

**Fig. 5:**
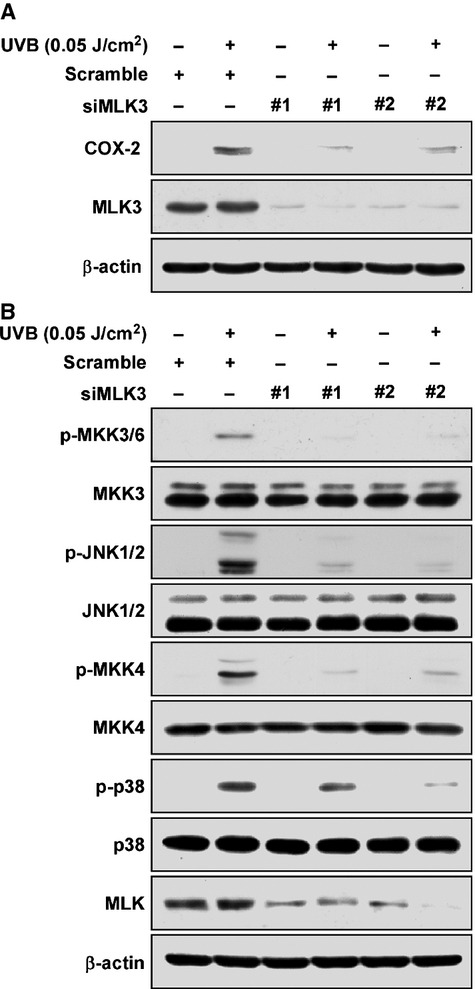
Effect of MLK3 knockdown on UVB-induced COX-2 expression, as well as MKK3/6 and MKK4 phosphorylation in HaCaT cells. (**A**) Knockdown of MLK3 reduces UVB-induced COX-2 expression in HaCaT cells. Expression levels of COX-2 were determined by Western blot assay with specific antibodies. (**B**) Knockdown of MLK3 reduces UVB-induced MKK3/6/JNK1/2 and MKK4/p38 phosphorylation in HaCaT cells. Phosphorylation was detected by Western blot assay with phosphor-specific antibodies. Data are representative of three independent experiments that gave similar results.

Knockdown of MLK3 significantly reduced UVB-induced COX-2 expression when compared with scrambled sequence controls (Fig. 5A). Additionally, siMLK3 inhibited UVB-induced MKK4/p38 and MKK3/6/JNK1/2 phosphorylation in HaCaT cells (Fig. 5B).

### DHGA-D inhibits UVB-induced COX-2 expression *in vivo*

**Fig. 6:**
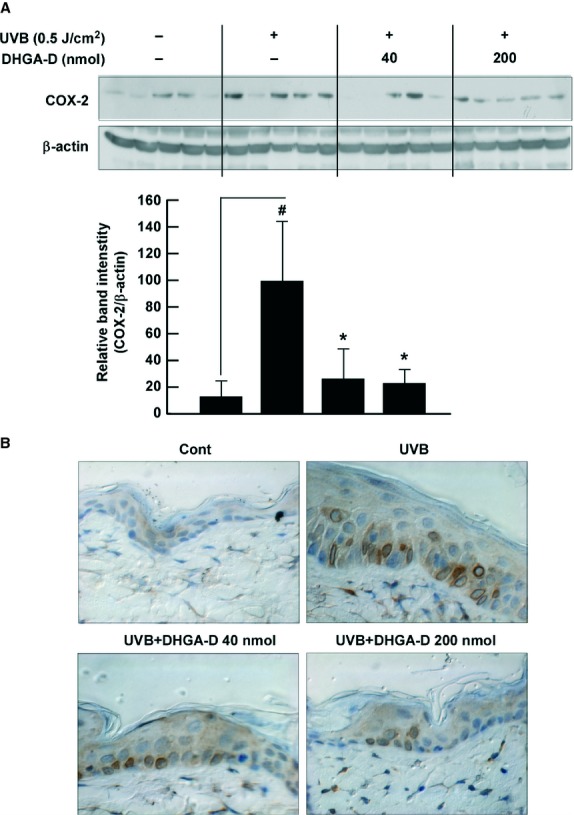
Effect of DHGA-D on UVB-induced COX-2 expression *in vivo*. (**A** and **B**) DHGA-D inhibits UVB-induced COX-2 expression in SKH-1 mouse skin. Expression levels of COX-2 and β-actin were determined by Western blot assay with specific antibodies. Each band was densitometrically quantified by image analysis. Results are shown as the means ± SEM (*n* = 5). The hash symbol (#) indicates a significant difference (*P* < 0.05) between the control group and the group exposed to UVB alone; asterisks (*) indicate a significant difference (*P* < 0.05) between groups irradiated with UVB and DHGA-D and the group exposed to UVB alone. In the immunohistochemical analysis, COX-2 is stained brown. Representative photographs of overall immunohistochemical staining patterns from each group are shown.

The SKH-1 mouse model is well suited for studying skin inflammation and chemoprevention using phytochemicals [3–5,30]. We used this model to investigate the effect of DHGA-D on UVB-induced COX-2 expression. Western blot assay results using mouse skin extract showed that DHGA-D significantly inhibited UVB-induced COX-2 expression *in vivo* (Fig. 6A). Our immunohistochemistry results also showed that DHGA-D suppressed UVB-induced COX-2 expression (Fig. 6B).

## Discussion

The regular consumption of natural phytochemicals has been closely linked to chemopreventive effects [12]. Both the NCI and NIH recommend higher consumption of natural foods, including vegetables and fruits [11]. Licorice has long been used as a traditional medicine and sweetener, particularly in East Asian cultures, and a number of its compounds have recently been identified as effective bioactive agents [13–18]. DHGA-D has been previously confirmed as an anti-obesity agent, as a result of its PPARγ ligand-binding activity [19]. Additionally, recent studies have reported that DHGA-D exhibits anti-aldose reductase and antioxidant effects [7,16]. However, its biological function in inflammation and chemoprevention, as well as the molecular mechanisms responsible, remains unclear. In the present study, we applied UV irradiation to HaCaT human keratinocytes and SKH-1 hairless mice to examine the effect and mechanism of DHGA-D.

*In vitro* and genetically modified animal study results have clearly shown that COX-2 is a critical player in inflammation and carcinogenesis, and COX-2 signalling is a promising target for chemoprevention [1,6,7,23,31]. In our previous studies, phytochemicals were shown to regulate abnormal COX-2 expression by targeting COX-2-regulatory signalling molecules [3–5,12]. Here, we found that DHGA-D completely inhibited UVB-induced COX-2 expression and PGE2 generation. In comparison with previous studies where the reported optimal concentration of phytochemicals was 20–50 μM for inhibition of COX-2 expression and kinase activity [3,5], DHGA-D showed higher efficacy at low doses (2.5 μM) and completely suppressed UVB-induced COX-2 expression and PGE2 production [3–5,17,32].

AP-1 is a core transcription factor in UV-induced inflammation and carcinogenesis. UVB irradiation is known to induce AP-1 expression [26,27]. Therefore, we determined the effect of DHGA-D on UVB-induced AP-1 transactivity. Luciferase assaying for the AP-1 promoter showed that DHGA-D significantly inhibited UVB-induced AP-1 transactivity, and accumulated evidence shows that MAPKs are critical regulators of such activity [32,33]. In our Western blot assay, DHGA-D strongly suppressed UVB-induced MKK3/6 and MKK4 signalling pathways without MLK3 phosphorylation (MKK3/6 and MKK4 are normally directly phosphorylated by MLK [8]). A previous study has shown that disruption of the murine *Mlk3* gene has been shown to result in selective reduction of JNK phosphorylation in response to TNF-α [9]. Additionally, SPLK (MLK3) activates p38 kinase in COS cells [28]. We therefore hypothesized that DHGA-D may directly inhibit MLK3 activity and lead to the suppression of MMK3/6 and MKK4. Kinase and immunoprecipitation assay results supported this hypothesis by showing that DHGA-D significantly suppresses MLK3 activity through direct binding to MLK3.

Although our results showed that DHGA-D suppressed UVB-induced COX-2 expression by directly inhibiting MLK3 activity and subsequent signalling including MAPKK/MAPK, to date, there has been no evidence to suggest that MLK3 regulates UVB-induced COX-2 expression. We found that knockdown of MLK3 induced down-regulation of UVB-induced MKK4/JNK1/2 and MKK3/p38 phosphorylation, resulting in suppression of COX-2 expression. These results indicate that MLK3 plays an important role in UVB-induced COX-2 expression by regulating MKK4 and MKK3 signalling. We further clarified that DHGA-D inhibited UVB-induced COX-2 expression in hairless mouse skin. Analysis of tissue samples confirmed that DHGA-D was able to inhibit UVB-induced COX-2 expression in mouse skin.

Taken together, our results suggest that DHGA-D significantly inhibits UVB-induced COX-2 expression *in vivo*. This inhibition occurs primarily through inhibition of the MLK3 kinase, leading to suppression of COX-2 expression through reduced MAPK and AP-1 activity. The therapeutic inhibition of MLK3 kinase by DHGA-D may provide clinical benefits for the better treatment of skin inflammation. This represents the first report to elucidate the molecular basis of the anti-inflammatory activity of DHGA-D.
